# Exploring cell death mechanisms in liver transplantation: implications for graft survival

**DOI:** 10.3389/fimmu.2025.1700382

**Published:** 2026-01-09

**Authors:** Hirofumi Hirao, Takeshi Watanabe, Yoichiro Uchida, Etsuro Hatano

**Affiliations:** 1 Department of Surgery, Division of Hepato-Pancreato-Biliary Surgery and Transplantation, Graduate School of Medicine, Kyoto University, Kyoto, Japan; 2 Laboratory of Immunology Institute for Life and Medical Sciences, Kyoto University, Kyoto, Japan

**Keywords:** cell death, damage-associated molecular pattern, innate immunity, ischemia-reperfusion injury, liver transplantation, sterile inflammation

## Abstract

Liver transplantation (LT) has become a life-saving therapy for patients with end-stage liver disease and malignancies. However, graft survival remains a significant challenge because of LT-related stresses. Grafts are subject to several stresses, including cold preservation after procurement and during transportation, as well as warm ischemia until vascular reconstitution, which can trigger hepatic cell death. This review examines the various cell death mechanisms that influence liver graft outcomes, including apoptosis, necrosis, autophagy, and non-apoptotic inflammatory cell death. We discuss how these mechanisms are driven by ischemia-reperfusion injury, which contributes to graft dysfunction. Apoptosis leads to the selective elimination of damaged hepatic cells, while necrosis, resulting from fulminant injury, can provoke inflammatory responses that further jeopardize graft viability. Autophagy emerges as a double-edged sword, promoting cellular repair under stress while potentially leading to cell death in extreme circumstances. Additionally, recent studies have uncovered novel non-apoptotic death pathways, such as necroptosis, pyroptosis, ferroptosis, panoptosis, and netosis, that may also influence transplant outcomes. Understanding the intricate interplay of these cell death mechanisms is vital for developing innovative therapeutic strategies to enhance graft survival. By synthesizing current research findings, this review aims to highlight the potential for targeted interventions to mitigate cell death and improve liver transplant outcomes, ultimately improving patient survival and quality of life.

## 1 Introduction

In liver transplantation, cell death plays a crucial role in determining the overall success and function of the transplanted organ, affecting graft survival and patient outcomes. The liver grafts experience significant cellular stress during the transplantation process due to two types of injury: warm ischemia and cold ischemia, which can be followed by reperfusion injury (1). These phases exert pathological effects on liver cell populations, including hepatocytes, liver sinusoidal endothelial cells (LSECs), and Kupffer cells (KCs). Each type of ischemic injury triggers distinct cellular responses, leading to various forms of cell death and ultimately impacting the graft after transplantation. First, the graft undergoes cold ischemia, a condition that occurs when the liver is preserved at low temperatures during storage and transportation after the donor liver is procured. Then, warm ischemia happens during the donor liver implantation till revascularization in the recipient. In the case of a donation after circulatory death (DCD) donor, the graft is subject to prolonged warm ischemia. These ischemic events contribute to ischemia-reperfusion injury (IRI), leading to early allograft dysfunction (EAD), a critical factor influencing liver transplantation outcomes (2, 3). IRI causes a surge of reactive oxygen species (ROS) and a strong inflammatory response. The ischemia stress causes hepatic glycogen consumption, oxygen deprivation, and ATP depletion. While hypothermia slows down metabolism and reduces immediate injury, prolonged cold storage induces specific types of sublethal cellular damage (4). These changes trigger the production of ROS and promote organelle damage, leading to hepatic cell death or injury, which ultimately releases damage-associated molecular patterns (DAMPs) from the liver (5). After reperfusion, released DAMPs activate host innate immune cells, evoking inflammatory cascades that further exacerbate graft damage (6). Several cell death pathways are activated during IRI, each with distinct mechanisms and effects on the graft. As our understanding of cell death mechanisms, such as necrosis, apoptosis, necroptosis, pyroptosis, ferroptosis, autophagy, panoptosis, and netosis, researchers are gaining insights into how these processes exacerbate or mitigate liver damage during transplantation. Although many types of cell death overlap and contribute during liver transplantation, there remains an incomplete understanding of the specific cell types involved and their associated cell death pathways, especially concerning warm and cold ischemia. Sterile inflammation, a hallmark of transplant rejection, is driven by DAMPs released from dying cells. When cells undergo pro-inflammatory cell death, DAMPs such as HMGB1, ATP, and mitochondrial DNA are released, which then activate pattern recognition receptors (PRRs) on immune cells, thereby initiating a cascade of inflammatory responses that recruit immune cells, such as neutrophils, macrophages, and T cells, to the injury site (6). Consequently, sterile inflammation can increase the risk of rejection, highlighting the need for strategies to control inflammatory responses following transplantation (7, 8). In this review, we summarize the current knowledge of the underlying mechanisms in cellular death during LT and highlight the potential for targeted interventions to protect liver grafts.

## 2 Hepatic cell susceptibility to warm and cold ischemia

**Figure 1:**
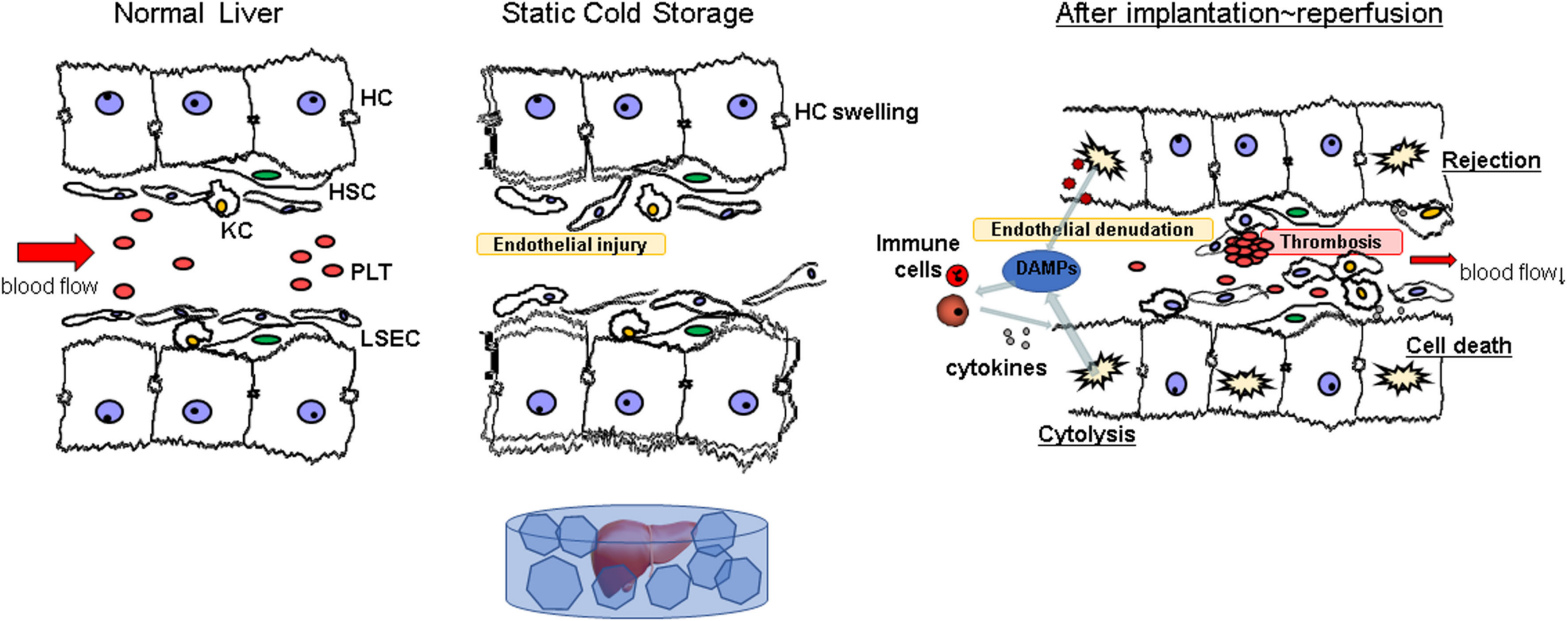
Schema of ischemia-reperfusion injury during liver transplantation. LSECs are susceptible to cold stress, leading to cell death during cold storage, which is called “cold ischemia”. Prolonged cold ischemic time sometimes causes hepatocellular swelling, which in turn narrows the vascular bed. After implantation to reperfusion, DAMPs released from dying cells recruit and activate immune cells, such as macrophages, neutrophils, and Kupffer cells, which in turn release pro-inflammatory cytokines, resulting in further hepatic damage. HC, hepatocyte; HSC, hepatic stellate cell; KC, kupffer cell; LSEC, liver sinusoidal endothelial cell; PLT, platelet.

The liver is composed of several cell types, each playing essential roles in its metabolic, immunological, and detoxifying functions. The major hepatic cell types include hepatocytes - the primary parenchymal cells, responsible for metabolism, protein synthesis, bile production, and detoxification; Liver sinusoidal endothelial cells (LSECs) - non-fenestrated endothelial cells lining the liver sinusoids, involved in filtration and substance exchange between blood and hepatocytes; Kupffer cells (KCs) - liver-resident macrophages that play a key role in innate immunity, phagocytosis, and cytokine production; Hepatic stellate cells (HSCs) - located in the space of Disse, involved in vitamin A storage and, upon activation, in liver fibrosis through extracellular matrix production; Cholangiocytes - epithelial cells that line the intrahepatic bile ducts and are responsible for bile modification and transport; Liver-associated natural killer (NK) cells - involved in intrahepatic immune surveillance; Liver dendritic cells - antigen-presenting cells contributing to immune tolerance and response regulation (9–11). In liver transplantation, both warm and cold ischemia contribute to graft injury, with distinct susceptibilities depending on the type of hepatic cell (6). Hepatocytes, while relatively more resistant to cold ischemia, are predominantly vulnerable to warm ischemia due to their high metabolic activity and oxygen consumption. Hepatocytes rapidly undergo ATP depletion, mitochondrial dysfunction, and oxidative stress, exhibiting marked functional impairment, including elevated transaminases and reduced bile flow. In contrast, LSECs are highly sensitive to cold ischemia and are the primary targets of cold preservation (12). Due to the delicate structure and poor antioxidant defenses, LSECs undergo cytoskeletal disruption, cellular swelling, early cellular death, and detachment during cold storage and reperfusion (13, 14). These changes predispose the liver to non-perfused areas (“no-reflow phenomenon”) after reperfusion (15), as illustrated in **Figure 1**. KCs exhibit impaired phagocytic function and are activated under both ischemic conditions, contributing to cytokine release and the recruitment of neutrophils, which leads to the exacerbation of tissue injury since pharmacological depletion of KCs attenuated warm and cold ischemia-induced inflammatory responses (13, 16, 17). KCs remain largely intact during cold ischemia but are potently activated upon reperfusion, while prolonged warm ischemia reduces the number of KCs, suggesting the susceptibility to warm ischemia (18). Although less studied, hepatic stellate cells may be involved in structural and microcirculatory alterations, particularly following cold ischemia, implying that HSCs might be vulnerable to cold ischemia (19). Since the pharmacological depletion or genetic suppression of HSCs proliferation attenuated IRI, HSCs may amplify IR-related inflammatory responses (20). Both cold and warm ischemia contribute to cholangiocyte injury by causing ATP depletion, the generation of reactive oxygen species (ROS), and damage to the peribiliary vascular plexus, leading to cholangiocyte necrosis and apoptosis. Indeed, prolonged donor warm ischemia time particularly increases the risk of ischemic cholangitis and biliary strictures due to damage to cholangiocytes and microvasculature (21). Additionally, prolonged cold ischemia time predisposes cholangiocytes to injury, especially in donation after circulatory death (DCD) grafts, by compromising bile duct microcirculation and promoting ROS-mediated damage (22). The role of DCs in warm injury in experimental settings is controversial, presumably depending on the subtypes of DCs. In warm IRI, depletion of conventional DCs worsens liver injury by less anti−inflammatory cytokines (e.g. IL−10) production and upregulation of IL−6/TNF production (23), whereas the ablation of plasmacytoid-DCs aggravated IR injury (24). In contrast, in a liver transplantation model, the ablation of donor DCs results in severe liver injury, elevated transaminases, graft necrosis, and neutrophil infiltration, highlighting the protective role of liver-resident DCs during cold preservation-related injury (25). However, little is known about the susceptibility of DCs to ischemic stress during warm and cold IRI. In a rat perfusion model, liver NK cells were released from the tissue into the perfusate in increasing numbers as the cold ischemia duration increased, suggesting mobilization and activation against ischemic injury (19). This result may imply a response to cold ischemia, whereas the impact of warm ischemia on NKs is less characterized. The vulnerability of different cell types also varies based on graft quality. Marginal grafts, such as steatotic livers, are more prone to ischemia-reperfusion injury due to increased lipid content or autophagy dysregulation, which sensitizes them to oxidative stress and inflammation, compromising graft survival (26). Thus, understanding these cellular susceptibilities is essential for developing cell-type-specific strategies to minimize ischemia-reperfusion injury in liver transplantation. Vulnerability of each hepatic cell type to warm and cold ischemia is summarized in **Table 1**.

**Table 1:** Susceptibility of liver resident cells to warm and cold stress.

Cell type	Warm	Cold
Hepatocyte	+++	+
Sinusoidal endothelial cell (LSEC)	+	+++
Kupffer cell (KC)	++	++
Hepatic stellate cell (HSC)	unknown	++
Cholangiocyte	++	++
Natural killer cell (NK)	unknown	+
Dendritic cell (DC)	unknown	unknown

## 3 Types of cell death in liver transplantation

### 3.1 Apoptosis

Apoptosis, a form of programmed cell death, is tightly regulated and occurs in a highly organized manner, which is believed to allow cells to die without triggering an inflammatory response (27). During apoptosis, the plasma membrane remains intact, preventing the release of intracellular damage-associated molecular patterns (DAMPs). Apoptotic cells exhibit cell shrinkage, chromatin condensation, and the formation of apoptotic bodies, which are later cleared by phagocytic cells without eliciting an inflammatory response (28). However, under certain pathological conditions, such as delayed clearance or excessive apoptosis, secondary necrosis may occur, potentially leading to inflammation (29). During liver transplantation, apoptosis is often observed in primarily hepatocytes and other liver cell types subjected to oxidative stress and hypoxia during ischemia. In liver grafts, apoptosis is likely to be less detrimental than other forms of cell death, as it does not release inflammatory intracellular contents. However, extensive apoptosis can still impair the overall function of the graft (30). A recent report documented that metabolites released from apoptotic cells could have a beneficial effect on surrounding tissues via a pannexin-1 (PANX-1) dependent manner (31). In addition, BAX/BAK-related apoptosis induced IL-1β maturation in macrophages through a caspase-8-dependent mechanism (32). Thus, in recent years, apoptosis - once referred to as a “silent” form of cell death - has increasingly been recognized as playing an active role in shaping the response of surrounding tissues and cells.

Apoptosis of mammalian cells can be categorized into intrinsic and extrinsic apoptosis. Extrinsic apoptosis is initiated by the engagement of death receptors on the plasma membrane, while intrinsic apoptosis is executed by DNA or mitochondrial damage (33). The intrinsic apoptosis is prominent during ischemia and early reperfusion. The ischemic phase leads to ATP depletion and ROS generation, priming hepatocytes for mitochondrial dysfunction. Upon reperfusion, oxidative stress can trigger mitochondrial outer membrane permeabilization (MOMP), resulting in the release of cytochrome c into the cytosol. Formation of the apoptosome by cytochrome c, Apaf-1 (apoptotic peptidase activating factor 1), and procaspase-9 activates caspase-9, which in turn mediates executioner caspase-3. The BCL-2 protein family plays a central role in intrinsic apoptosis and is the key regulator of MOMP. BCL-2, BCL-xL, and MCL-1 serve as the anti-apoptotic proteins, while pro-apoptotic BAX and BAK drive MOMP. Several reports have documented that the engagement of Bcl-2 protein expression alleviated liver damage in experimental warm IRI (34, 35) and LT (36) models. Also, the pharmacological modulation of intrinsic apoptosis, including mitochondrial stabilizers such as cyclosporine A, has been shown to attenuate hepatic injury in experimental models (37–41).

The extrinsic pathway is activated by death ligands binding to death receptors, especially during the reperfusion phase when immune cells infiltrate the liver. Pro-inflammatory cytokines such as TNF-α, FasL, and Tumor necrosis factor-related apoptosis-inducing ligand (TRAIL) released by infiltrating immune cells, engage their respective death receptors (TNFR1, Fas, DR4/5). Death receptors such as Fas (CD95), TNF receptor 1 (TNFR1), and TRAIL receptors are expressed on hepatocytes, Kupffer cells, and non-parenchymal cells (42, 43). Upon ligand binding, these receptors trigger the formation of the death-inducing signaling complex (DISC), recruit adaptor proteins (FADD, TRADD), and activate caspase-8, leading to downstream caspase-3 activation and apoptotic cell death. Caspase-8 can also cleave BID to tBID, linking to the mitochondrial (intrinsic) pathway. Experimental studies have shown that ischemia triggers hepatocyte sensitization to Fas-mediated apoptosis, and blocking the Fas-FasL interaction reduces liver injury in the rat IRI model (44, 45) and allotransplantation (46). In contrast, another report documented that the genetic ablation of Fas or FasL in mice exhibited comparable liver injury with the wild-type (WT) counterpart, suggesting that Fas signaling was not necessary to mediate IR-stress-induced hepatic apoptosis (47). The explanations to reconcile these conflicting results may be the difference in species, ischemia type, and timing. Necrosis may be dominant during the acute warm ischemia phase (48), while Fas–FasL may become more relevant in the reperfusion/inflammatory phase or in cold ischemia during transplantation. Kupffer cells release TNF-α during reperfusion, which not only promotes inflammation but also induces hepatocyte apoptosis via TNFR1 signaling. Inhibition of TNF-α/TNFR1 signaling ameliorates hepatocellular damage in rodent models of warm IRI (49). Notably, in the clinical transplantation setting, TRAIL expression was down-regulated in both the donor graft and allograft (50). Also, depletion of TRAIL in a murine model of warm IRI augmented IR-related injury, and adoptive transfer of TRAIL-positive NK cells ameliorated liver damage (51). These findings suggest that the role of TRAIL may vary depending on the cell type, reflecting cell-specific sensitivity to death receptor signaling and the surrounding microenvironment.

### 3.2 Necrosis

Unlike apoptosis, necrosis is characterized by an uncontrolled form of cell death where prolonged oxygen deprivation, ATP depletion, mitochondrial failure, and excessive reactive oxygen species (ROS) lead to cellular energy collapse and transition from reversible injury to irreversible necrotic death, thereby releasing the cellular contents, including damage-associated molecular patterns (DAMPs) such as HMGB1, ATP, nuclear proteins, and mitochondrial DNA. These molecules are rapidly sensed by pattern-recognition receptors (TLRs, NLRs) on Kupffer cells, infiltrating monocyte-derived macrophages, and neutrophils, triggering a robust inflammatory response that activates innate immune cells and exacerbates liver damage. During liver transplantation, necrosis represents a central driver of sterile inflammation, a host-derived inflammatory process occurring in the absence of infection. Cold and warm ischemia synergistically exacerbate these processes, and reperfusion introduces sudden oxidative stress and calcium influx, further promoting membrane breakdown. As a consequence, necrotic burden correlates with peak transaminase release, microvascular dysfunction, and early allograft dysfunction in both clinical and experimental models, which markedly influences early graft function and can potentiate subsequent rejection as discussed later. Importantly, necrosis is more prominent in grafts with pre-existing vulnerability. Steatotic or marginal livers exhibit impaired mitochondrial resilience, increased lipid peroxidation, and reduced antioxidant capacity, making them disproportionately prone to necrosis during ischemia (52). In such cases, even relatively short ischemic duration can precipitate massive necrotic injury, explaining the increased rates of early allograft dysfunction, biliary complications, and lower survival observed clinically. Therefore, limiting necrotic cell death through organ preservation techniques, mitochondrial-protective agents, or modulation of iron-dependent oxidative injury is considered a promising strategy to improve liver transplant.

### 3.3 Necroptosis

Necroptosis is a form of regulated necrotic cell death mediated by the RIPK1–RIPK3–MLKL axis, triggered when death receptors (e.g., TNFR1, Fas) are engaged without caspase−8 activation. RIPK1 interacts via its RHIM domain with RIPK3, leading to necrosome assembly, RIPK3 autophosphorylation, and activation of MLKL. Phosphorylated MLKL oligomerizes and translocates to cellular membranes, causing lytic rupture and release of DAMPs such as HMGB1, cytokines (TNF−α, IFNs), and intracellular contents, thereby amplifying inflammation (53). Recent studies implicate necroptosis as a critical mediator of liver ischemia–reperfusion injury, particularly under pathophysiological conditions such as steatosis or ageing (54). In murine models of fatty liver (Western diet–induced steatosis), IR injury was exacerbated, associated with elevated RIPK3 and MLKL expression and increased necrotic hepatocyte death (55, 56). Genetic ablation of MLKL (57) or myeloid RIPK3 (58), or pharmacological inhibition of RIPK1 (e.g., Necrostatin−1) or RIPK3 (e.g., GSK′872) (59), significantly attenuates hepatocellular death, inflammatory cytokine production, and histological liver damage in murine IR models. However, the protective effect targeting necroptosis was limited when treated with WT mice (60). These findings suggest that necroptosis is a viable therapeutic target for reducing hepatic injury in transplantation and acute liver failure, especially in fatty or aged livers. Given that necroptosis is augmented in steatohepatitis in both mouse and human (61, 62), targeting necroptosis may be more effective in marginal grafts (e.g., steatosis).

### 3.4 Pyroptosis

Pyroptosis is a lytic form of programmed cell death associated with inflammasome activation (NLRP3) and caspase-1/11, which results in the cleavage of gasdermin D (GSDMD), leading to the formation of membrane pores that release pro-inflammatory cytokines such as IL-1β and IL-18 (63). The inflammasome, a multiprotein complex activated by cellular stress, initiates pyroptosis primarily in response to DAMPs and pathogen-associated molecular patterns (PAMPs). Other gasdermin family proteins, such as GSDMA, GSDMB, GSDMC, and GSDME, are activated by inflammasome-independent mechanisms to induce pyroptosis (64). In liver grafts, pyroptosis contributes to inflammation and liver damage following ischemia-reperfusion, further complicating the immune response and increasing the risk of graft rejection (65, 66). Pyroptosis has been extensively reported in immune cells, particularly in macrophages (67), where inflammasome activation and gasdermin-mediated pore formation are central mechanisms. In the context of liver ischemia-reperfusion injury (IRI) and transplantation, macrophage pyroptosis, including that of liver resident macrophages, such as Kupffer cells (68), contributes to the amplification of inflammatory cascades through the release of IL-1β, IL-18, and damage-associated molecular patterns (DAMPs) (69). These mediators further recruit and activate neutrophils and other immune cells, exacerbating hepatic injury. Recent studies also suggest that donor-derived macrophages undergoing pyroptosis may influence acute graft versus host disease (70), indicating that pyroptosis is not merely a bystander phenomenon but an active driver of inflammation and tissue damage in liver IRI. In hepatic IRI models, activation of the NLRP3 inflammasome in macrophages, particularly Kupffer cells, leads to caspase-1–mediated cleavage of gasdermin D (GSDMD), resulting in pore formation, release of IL-1β/IL-18, and amplification of local inflammation (71). Importantly, myeloid-specific GSDMD deletion or pharmacologic inhibition of caspase-1 significantly attenuates hepatic IRI and inflammatory cytokine release. In contrast, hepatocyte-specific GSDMD deletion does not confer protection, although hepatocytes underwent GSDMD-mediated pyroptosis in an XBP1-dependent mechanism in the drug-induced acute liver injury model (72). These results underscore the dominant role of innate immune cell pyroptosis over parenchymal cell death in exacerbating IRI (69). Recent mechanistic studies further reveal that mitochondrial protection occurs in liver macrophages. Maresin 1, can suppress pyroptosis, down−regulating IL−1β/IL−18 release via activation of RORα and PI3K/AKT signaling, and ameliorate liver IRI, highlighting its therapeutic potential (73). Quercetin, a flavonoid, inhibited macrophage pyroptosis by blocking GSDMD cleavage and disrupting Caspase−8/ASC complex formation (74). Glycyrrhizin suppressed GSDMD−mediated pyroptosis in Kupffer cells through inhibition of HMGB1−dependent signaling, leading to reduced IL−1β release and liver tissue damage (75). Piceatannol, a resveratrol derivative, prevented macrophage pyroptosis by inhibiting the TLR4–NF−κB–NLRP3 cascade, reducing caspase−1 activation, IL−1β/IL−18 production, and GSDMD−N formation in hepatic IRI model (76). Additionally, the upstream signaling axis of TLR4/MyD88/NF-κB-to-NLRP3 contributes to macrophage pyroptosis during warm IRI, and pharmacologic blockade of MyD88 (e.g., with TJ-M2010-5) reduces pyroptosis and improves hepatic outcomes (77). Emerging regulatory mechanisms include the Ikaros-Sirtuin1 (SIRT1) signaling axis within liver-infiltrating macrophages, which modulates canonical inflammasome activation and consequent pyroptosis in both experimental IRI and human transplant biopsies (78). Furthermore, system-level reviews of hepatic macrophage pyroptosis in liver disease reinforce its central role in driving inflammation, fibrosis, and immune-mediated injury, establishing it as a compelling therapeutic target across a range of liver pathologies (71). In contrast, Caspase−1 activation and GSDMD cleavage were significantly elevated following IRI in a steatotic (high−fat diet) mouse model. Knockout of Caspase−1 or Caspase−1/11, not Caspase-11 alone, markedly reduced serum ALT, histologic injury scores, and hepatocellular death, supporting hepatocyte pyroptosis as a key driver of injury in fatty livers (79). Similarly, SIRT1-deficient hepatocytes are susceptible to cold stress and prone to GSDME-dependent pyroptosis in mouse and human liver transplantation (80). Accumulating evidence indicates that pyroptosis, mediated via the NLRP3 inflammasome and GSDM-dependent mechanisms, significantly contributes to liver ischemia–reperfusion injury. Thus, macrophage pyroptosis is likely to be prominent, while hepatocytes can also execute pyroptosis under specific conditions. Targeting pyroptosis pathways-both canonical and non-canonical-offers a promising therapeutic approach for reducing inflammatory liver damage during surgery or transplantation.

### 3.5 PANoptosis

PANoptosis is an inflammatory programmed cell death (PCD) paradigm in which the molecular machinery of apoptosis, pyroptosis, and necroptosis is co-activated and coordinated by multicomponent “PANoptosome” complexes assembled by innate immune sensors/adaptors (e.g., ZBP1-, AIM2-, RIPK1-, or NLRP12-PANoptosomes). Core nodes include CASP8/FADD/RIPK1/RIPK3/MLKL (the apoptosis-necroptosis axis) and ASC/CASP1/GSDMD (the pyroptosis axis), with crosstalk enabling simultaneous or sequential execution across these pathways (81, 82). Compared with single-pathway programmed cell death, PANoptosis amplifies inflammation (IL-1β/IL-18 release via pyroptosis), membrane lysis (pyroptosis/necroptosis), and apoptotic fragmentation, thereby worsening hepatocellular and sinusoidal endothelial injury and sterile inflammation (83). It is not surprising that liver IRI can activate sensors (e.g., ZBP1, STING) and signaling nodes (e.g., TAK1, RIPK1/RIPK3, caspase-8) that can assemble PANoptosomes and execute blended death programs in hepatocytes and non-parenchymal cells since IR stresses create a burst of DAMPs (ROS, mtDNA), cytokines, and pattern-recognition signaling that can trigger PANoptosome assembly in the liver. Hepatic I/R promotes mitochondrial damage and cytosolic mtDNA release. Stimulator of interferon genes (STING) activation has been shown to exacerbate PANoptosis in hepatocytes during IRI. Pharmacologic STING inhibition by IMT1B mitigated PANoptosis and liver damage in preclinical IRI models, nominating the pathway as a tractable target (84). In endothelial cells, IRI was shown to induce ZBP1-dependent PANoptosis. Hypoxia/reoxygenation triggers PANoptosome assembly via ZBP1, and silencing ZBP1 suppresses PANoptotic cell death, highlighting a potential mechanistic parallel relevant to vascular damage in grafts (85). On the contrary, previous work demonstrated that LSECs were highly sensitive to cold stress and readily initiated ferroptotic cell death (12). Taken together, there might be a crosstalk between Panoptosis and ferroptosis. More in-depth mechanistic explorations are warranted. In fatty liver I/R (high-risk in transplantation), ROS-driven ZBP1 aggregation activates RIPK1 to drive apoptosis and inflammation independent of Z-nucleic acid sensing, aggravating injury. While this study primarily documents RIPK1-dependent apoptosis/inflammation, ZBP1 is a canonical PANoptosis scaffold in other settings (85), implicating this node as an upstream PANoptotic trigger in steatotic graft vulnerability. Caspase-8 integrates death-receptor apoptosis, licenses or restrains necroptosis (via RIPK3/MLKL), and can engage pyroptotic execution through gasdermin cleavage under specific inflammatory contexts-thus positioning caspase-8 centrally in hepatic sterile inflammation and potential PANoptosis during I/R (86). A recent bioinformatics-driven study identified PANoptosis-related biomarkers predictive of hepatic IRI following liver transplantation. Six genes, including CEBPB, HSPA1A, HSPA1B, IRF1, SERPINE1, and TNFAIP3, were associated with IRI and regulated by NF-κB and miRNA-155, suggesting that PANoptosis could underlie graft injury mechanisms and that combined death-pathway activation is detectable in clinical samples, potentially helping to stratify early allograft dysfunction (87). Definitive *in vivo* mapping of PANoptosis across hepatocytes vs. LSECs vs. Kupffer cells during human reperfusion is outstanding. Single-cell and spatial proteogenomics in peri-reperfusion biopsies could clarify where PANoptosomes assemble. Beyond STING-driven hepatocyte PANoptosis, more genetic dissection (cell-specific deletion of caspase-8, GSDMD, MLKL, ZBP1) in liver I/R is needed to establish necessity/sufficiency. Given ZBP1-RIPK1 activation and the high clinical burden of MASLD donors, whether steatosis preferentially bias toward PANoptosis during I/R awaits future study.

### 3.6 Ferroptosis

Ferroptosis is an iron-dependent form of cell death characterized by lipid peroxidation, the accumulation of ROS, and mitochondrial membrane damage (28). Recent evidence underscores the central role of ferroptosis in exacerbating hepatic injury during ischemia–reperfusion and transplantation. Notably, LSECs are uniquely vulnerable to cold preservation–induced ferroptosis, particularly when NF-E2-related factor 2 (NRF2) signaling is compromised. Suppression of mitochondrial calcium uptake 1 (MICU1) or the use of ferroptosis inhibitors (Fer-1) affords cytoprotection against cold stress and improves graft outcomes (12). In liver IRI, the following hallmarks are consistently observed: increased iron accumulation, elevated prostaglandin-endoperoxide synthase 2 (PTGS2; ferroptosis marker), downregulation of glutathione peroxidase 4 (GPX4) and system Xc-components (e.g. SLC7A11), and ultrastructural mitochondrial changes such as cristae loss and high electron density (88, 89). Clinical data from pediatric living donor liver transplantation showed that high donor serum ferritin (iron overload marker) independently predicted worse post-transplant IRI outcomes. Elevated lipid peroxidation and PTGS2 expression in mouse IRI were also mitigated by ferrostatin−1 (Fer−1) or α-tocopherol, and aggravated by iron overload but alleviated by deferoxamine (iron chelation) (90). In mouse and human liver transplantation, Fer-1 treatment significantly reduced hepatic necrosis area and serum ALT/AST, reversed upregulation of TFR1 and ACSL4, and preserved GPX4 and FTH1 levels, while suppressing mitochondrial damage typical of ferroptosis (91). Fucoidan, a sulfated polysaccharide, prevented ferroptosis by enhancing Nrf2 nuclear translocation and downstream expression of HO−1 and GPX4, thereby reducing iron accumulation, lipid Dimethyl fumarate exhibited protective effects via activation of the Nrf2/SLC7A11/HO-1 axis, suppressing ferroptosis in hepatic IRI contexts (92, 93). RACK1 (Receptor for Activated C Kinase 1) was found to exert endogenous protection by interacting with AMPKα and promoting its phosphorylation at Thr172. Loss of RACK1 or its degradation via lncRNA ZFAS1 aggravated ferroptosis in hepatocytes under IRI stress, highlighting potential therapeutic or prognostic targeting (94). A direct comparison of cell death pathways in steatotic liver IRI demonstrated that among apoptosis, necroptosis, pyroptosis, and ferroptosis, ferroptosis inhibition (Fer-1) provided the most robust protection. In this model, ferroptosis primarily affected hepatocytes, while pyroptosis occurred mainly in macrophages (95). In steatotic livers, ferroptosis predominates in hepatocytes, and its inhibition yields stronger protection compared to blockers of apoptosis or necroptosis. Thus, ferroptosis appears to be a promising target for therapeutic strategies to reduce hepatic injury in transplantation and resection, especially under conditions of metabolic stress or iron overload.

### 3.7 Autophagy

**Figure 2:**
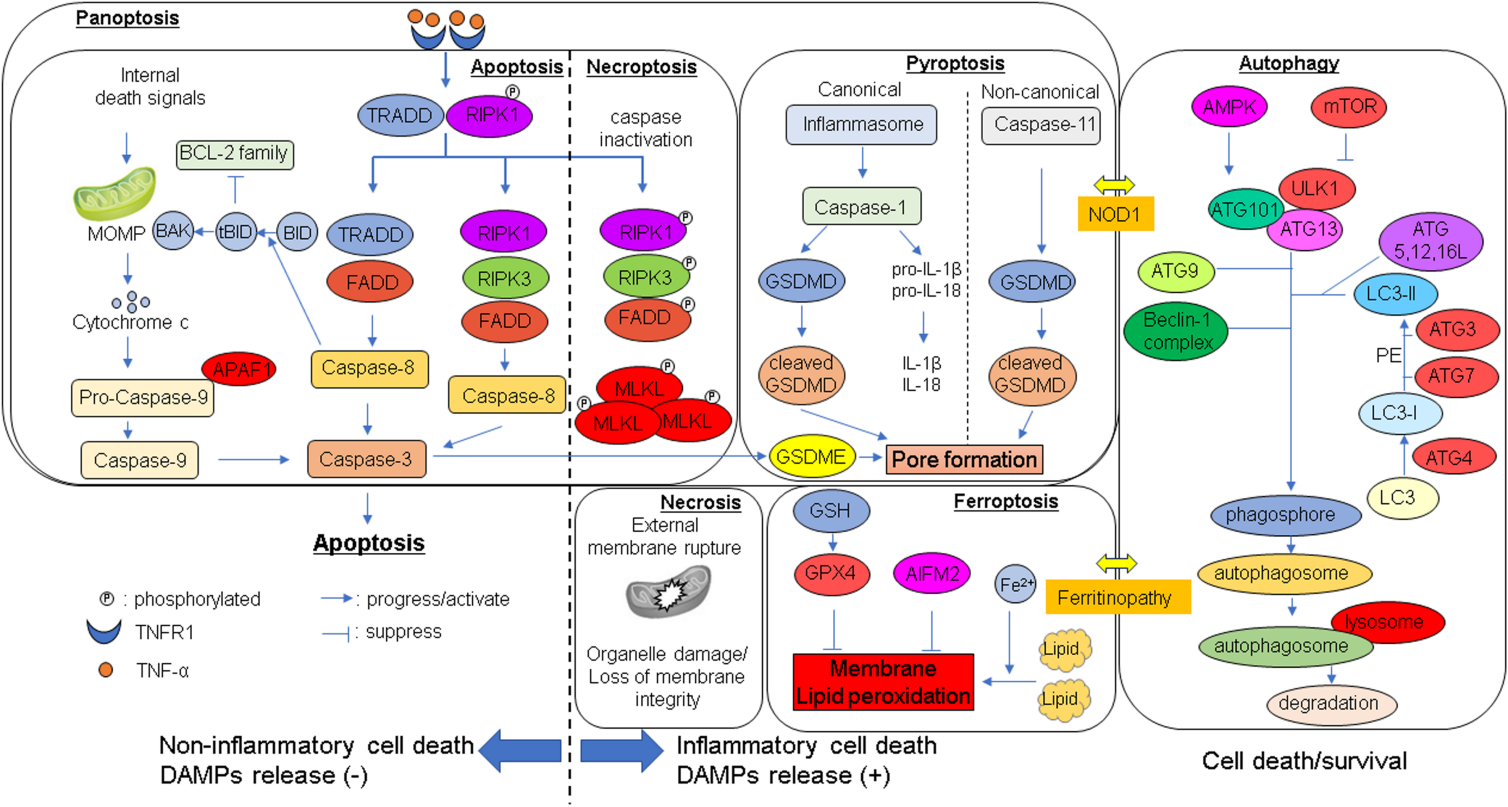
Molecular mechanisms of cell death in liver ischemia-reperfusion injury. Cellular signaling pathways are depicted in apoptosis, necroptosis, pyroptosis, panoptosis, ferroptosis, and autophagy. Each mode of cell death is triggered by its own distinct signaling pathway. Under certain conditions, it can also induce alternative forms of cell death. For example, caspase-3, which is essential for apoptosis, can cleave gasdermin E and thereby trigger pyroptosis. Similarly, NOD1 can induce pyroptosis but also promote autophagy-mediated cell death. In addition, autophagy can amplify ROS production, leading to the induction of ferroptosis. AIFM2, apoptosis inducing factor mitochondrion associated 2; AMPK, AMP-activated protein kinase; ATG, autophagy-associated gene; BAK, Bcl-2 homologous antagonist/killer; BCL, B-cell/CLL lymphoma 2; BID, BH3-interacting domain death agonist; FADD, Fas-associated protein with death domain; GPX4, glutathione peroxidase 4; GSDMD, gasdermin D; GSDME, gasdermin E; GSH, glutathione; LC3, Microtubule-associated protein light chain 3; MLKL, Mixed lineage kinase domain-like; MOMP, mitochondrial outer membrane permeabilization; mTOR, mammalian target of rapamycin; NOD1, Nod-like receptor 1; PE, phosphatidylethanolamine; RIPK, receptor interacting protein kinase; TRADD, TNFR-associated death domain; ULK1, unc-51 like autophagy activating kinase 1.

Autophagy is a conserved cellular degradation process that removes damaged organelles and proteins through lysosomal pathways. Autophagy generally consists of six steps: initiation, nucleation, elongation, maturation, fusion, and degradation (96). In the context of hepatic IRI and LT, autophagy plays a dual role, acting as both a protective mechanism and a contributor to cell death depending on the extent and duration of activation, and the impact of autophagy on IR-related stress is controversial. During the ischemic phase, reduced ATP levels activate AMPK and suppress mTOR, leading to ULK1 activation and autophagy initiation (97). Upon reperfusion, ROS generation and BNIP3 expression promote the release of Beclin-1 from Bcl-2, thereby enhancing autophagosome formation (98). By degrading damaged cellular components and recycling nutrients, autophagy can help maintain cellular homeostasis and reduce cellular stress. This process helps remove dysfunctional mitochondria, reduce ROS production, and attenuate inflammation, thereby protecting grafts from hepatic cell death (99). Post-reperfusion, increased levels of LC3-II, Beclin-1, and p62/SQSTM1 are commonly used to assess autophagic activity in liver tissue (100). Moderate autophagic activity clears damaged organelles and reduces oxidative stress, particularly during early ischemia through AMPK-mediated inhibition of mTOR and ULK1 activation (101). However, overactivation of autophagy leads to uncontrolled degradation of mitochondria and essential cellular components, ultimately resulting in cell death. Hepatocytes are the most well-studied cells in autophagy since hepatocytes are closely related to energy metabolism in the liver. Autophagy in LSECs is protective driven by ROS-mediated activation of ATG7 and formation of autophagosomes that clear damaged components. Disruption of autophagic flux in LSECs leads to endothelial necrosis and worsened IRI (102). In addition, selective mitophagy limits mitochondrial ROS generation, thereby preventing secondary cellular injury (103). Autophagy in Kupffer cells (104) and dendritic cells (105) also regulates inflammatory cytokine production, contributing to the modulation of innate immune responses. Increased Stat3 expressions are associated with ATG5 and anti-apoptotic effect against liver IRI, while suppression of stat3 or autophagy inhibitor, bafilomycin A1, aggravated liver IRI (106). On the contrary, when autophagic activity is excessive or dysregulated, especially under steatotic or aged liver conditions, it can lead to autophagic cell death, depleting essential cellular components and energy reserves. This excessive autophagy can exacerbate liver injury by increasing liver damage, leading to worse outcomes in liver IR injury. In experimental models, inhibition of autophagy by modulating the GLP-1 receptor with Exendin-4 has been shown to retain mitochondrial structure and attenuate hepatocellular injury in steatotic murine IRI (107). In addition, NOD1, an intracellular pattern recognition receptor, augmented autophagy activity to aggravated liver IRI, and silencing NOD1 attenuated IR injury ATG5 dependent manner (108). Inducers such as rapamycin enhances protective autophagy during ischemic stress through Beclin-1 and ATG4B upregulation, whereas inhibitors like chloroquine abolished the protective effect of autophagy induction (109). In aged rat models of IRI, young plasma restored aging-related impaired autophagy through the AMPK-ULK1 signaling pathway (110). Upregulation of IRF-1 leads to activation of the JNK signaling pathway, which in turn induces Beclin-1 expression and excessive autophagy, exacerbating liver injury. Suppression of IRF-1 demonstrated protective effects against IRI (111). Administration of umbilical cord-derived mesenchymal stem cells (UC-MSCs) activated AMPKα and promoted PINK1-mediated mitophagy, thereby stabilizing mitochondrial function and improving hepatocyte survival in IRI models (112). In a rat model of hepatic IRI, treatment with L-NAT suppressed the upregulation of LC3-II, Beclin-1, and ATG-7, while restoring p62 levels, thereby inhibiting excessive mitophagy and demonstrating hepatoprotective effects (113). Thus, while autophagy serves as an adaptive response in the early phase of IRI, its overactivation may paradoxically enhance tissue injury rather than prevent it. Taken together, autophagy exerts a context-dependent influence in liver transplantation and IRI. Moderate activation is hepatoprotective during ischemia, whereas excessive activation may worsen reperfusion injury. A deeper understanding of the temporal dynamics and cell-specific regulation of autophagy is crucial for developing targeted therapies that improve liver graft outcomes. Moreover, autophagy intersects with other regulated cell death pathways, including apoptosis, necroptosis, and ferroptosis, highlighting its central position in the pathophysiology of liver IRI. Understanding the precise thresholds at which protective autophagy shifts toward autophagic cell death remains a critical challenge, with significant implications for the development of therapeutic strategies in clinical liver transplantation. Therefore, the therapeutic application of autophagy modulation requires precise temporal and cellular control. The schema of aforementioned molecular pathways in cellular death is illustrated in **Figure 2**.

### 3.8 Netosis

Netosis is a specialized form of cell death in neutrophils, where they release their chromatin to form neutrophil extracellular traps (NETs), which help trap pathogens. However, during liver transplantation, NETs can accumulate and contribute to inflammatory damage in the graft, amplifying ischemia-reperfusion injury. Although netosis plays a role in immune defense, excessive NET formation can lead to tissue damage and worsen graft outcomes. Neutrophil extracellular traps (NETs) are emerging as pivotal mediators of hepatic IRI. During reperfusion, hepatocyte- and endothelial-derived DAMPs such as HMGB1 and histones stimulate neutrophils via TLR4/TLR9 and ROS signaling to undergo NETosis. NETs further promote immunothrombosis by binding platelets and facilitating microthrombus formation in the liver and distant organs, exacerbating tissue injury (114, 115). NET components, including CitH3 and neutrophil elastase, also exert direct cytotoxic effects. Experimental interventions—such as DNase I administration, PAD4 blockade, antioxidant therapies (e.g., N-acetylcysteine), curcumin treatment, and recombinant thrombomodulin—effectively mitigate liver injury by suppressing NET formation and its downstream effects. Given the involvement of NETosis in graft dysfunction, thrombosis, and potentially cancer recurrence, targeting NET-related pathways offers a promising therapeutic avenue in perioperative management of hepatic IRI in transplantation and hepatic surgery. The role of NETosis in liver pathology, including IRI, is well summarized elsewhere (116, 117).

### 3.9 Cell type-specific susceptibility to programmed cell death in liver transplant ischemia-reperfusion

Thus, warm and cold IRI during LT exhibits distinct patterns of cell death across hepatic cell populations, reflecting differences in metabolic demand, microcirculatory disturbance, and immune activation. As summarized in **Table 2**, hepatocytes are susceptible to necrosis and ferroptosis during warm IRI (48, 90, 118), driven by rapid ATP depletion and calcium overload. In hepatocytes, mitochondrial injury and oxidative stress accumulated during cold ischemia serve as a priming process, leading to more severe inflammatory cell death after reperfusion. Furthermore, the observation that iron chelation during cold storage attenuates post-reperfusion inflammatory cell death suggests that hepatocytes are particularly susceptible to ferroptosis and necrotic death during warm ischemia (119). However, a recent report revealed that hepatocytes can undergo pyroptosis via the Sirt1/Gasdermin E axis (80). In parallel, cold storage induces lipid peroxidation and iron-dependent damage, rendering LSECs prone to ferroptosis (12). Also, cold stress impairs autophagic flux, thereby leading to autophagic cell death via a Krüppel-like factor 2-dependent mechanism after reperfusion (120). Moreover, LSECs could undergo early apoptosis and detachment during warm ischemia, driven by heightened ROS sensitivity and shear stress-related injury (121). KCs exhibit a shift toward pro-inflammatory activation rather than cell death during the early phase; however, a prolonged warm ischemic insult can trigger pyroptosis via NLRP3 inflammasome signaling and necroptosis, while cold stress impairs phagocytic activity. Similarly, warm IRI can induce necroptosis and pyroptosis in BMDMs following inflammatory activation, thereby amplifying tissue damage (60, 122). Cholangiocytes are vulnerable to both warm and cold ischemia due to their dependence on peribiliary vascular plexus perfusion, leading to apoptosis, senescence-like phenotypes, and late-stage necrosis, which contribute to biliary complications post-transplantation (21). Single-cell studies have demonstrated upregulation of injury-related genes in cholangiocytes under cold IRI conditions (122). Together, understanding cell-type-specific vulnerability and preferential death pathways provides insight into therapeutic targeting strategies tailored to the ischemic context and graft condition.

**Table 2:** Differential susceptibility of liver cells to death pathways in warm and cold IRI.

Cell type	Cold	Warm
Hepatocyte	Pyroptosis	Apoptosis/Necrosis/Ferroptosis/Necroptosis (steatosis)
LSEC	Ferroptosis	Apoptosis/Autophagy
KC	phagocyte↓	Apoptosis/Necroptosis/Pyroptosis
BMDM	NA	Necroptosis/Pyroptosis
Cholangiocyte	Apoptosis/Necrosis	Apoptosis/Necrosis

NA, not applicable

## 4 How IRI contributes to graft rejection in liver transplantation

IRI promotes graft rejection by converting the transplanted liver from a relatively tolerogenic organ into a highly immunogenic phenotype through a series of interconnected immunological and microvascular processes (123). In the early phase of reperfusion, the aforementioned regulated and unregulated cell death results in the release of abundant danger-associated molecular patterns (DAMPs), such as HMGB1, ATP, and mitochondrial DNA (124, 125). These molecules are rapidly sensed by pattern-recognition receptors on Kupffer cells, dendritic cells, neutrophils, and complement components, initiating a cascade of innate immune activation. The resulting production of inflammatory cytokines and chemokines (e.g., TNF-α, IL-1β, IL-6, CXCL1/2) establishes a strongly pro-inflammatory microenvironment within the graft, effectively lowering the threshold for subsequent adaptive immune activation and rejection (124). Simultaneously, LSECs, which play a central role in maintaining microvascular tone and immune quiescence, promote the upregulation of adhesion molecules such as ICAM-1 and VCAM-1 in response to IRI, together with increased expression of MHC class I and II molecules and co-stimulatory ligands (126, 127). These phenotypic changes facilitate efficient leukocyte recruitment, enhance antigen presentation capacity, and reinforce both direct and indirect pathways of alloantigen recognition. Consequently, activated T cells gain easier access to the parenchyma, accelerating the onset and amplification of acute cellular rejection (128). Complement activation acts as an additional amplifier of the inflammatory cascade. The generation of C3a and C5a promotes neutrophil chemotaxis and activation, while the formation of the membrane attack complex (MAC) directly damages hepatocytes and endothelial cells. Crosstalk between the complement and coagulation systems further contributes to microvascular dysfunction, platelet aggregation, and sinusoidal thrombosis, thereby increasing the severity of IRI and creating a tissue environment conducive to sustained alloimmune injury (5). Beyond parenchymal damage, biliary complications represent a critical chronic consequence of IRI. Cold ischemia particularly affects cholangiocytes and the delicate peribiliary vascular plexus, leading to apoptosis, necrosis, and impaired bile duct regeneration. Over time, this ischemic insult may progress to ischemic cholangiopathy, biliary stricturing, and fibro-obliterative changes characteristic of chronic ductopenic rejection, ultimately impacting long-term graft survival (22). Dendritic cells serve as a crucial link between innate damage signals and adaptive immune rejection. Initially tolerogenic under homeostatic conditions, hepatic DCs undergo maturation in response to DAMPs, oxidative stress, and pro-inflammatory cytokines. Mature DCs acquire enhanced antigen presentation capacity, migrate to lymphoid tissues, and activate alloreactive CD4^+^ and CD8^+^ T cells. This transition represents a pivotal turning point at which sterile reperfusion injury is translated into a sustained adaptive alloimmune response (129). Collectively, these processes illustrate how IRI acts as a central immunological trigger that transforms the graft environment and substantially increases the risk of both acute and chronic rejection. Consequently, therapeutic strategies aimed at minimizing IRI, preserving endothelial integrity, and modulating DAMP-driven inflammation hold strong potential to improve post-transplant outcomes.

## 5 Targeting IRI in liver transplantation - the potential of machine perfusion to overcome IRI

In both experimental animal models and clinical settings, numerous strategies have been explored to mitigate IRI in liver transplantation. These include pharmacological interventions, ischemic preconditioning, and other protective approaches, with several recent studies providing comprehensive overviews of their efficacy (130, 131). However, as noted above, multiple modes of cell death can occur simultaneously within a single hepatic cell population during warm or cold ischemia, suggesting that a tailored, multimodal therapeutic strategy combining different interventions may be required rather than a single-target approach. Another emerging avenue is the growing use of machine perfusion technologies, which reduce or even eliminate ischemic exposure itself and therefore represent a promising alternative strategy for IRI prevention. Although static cold storage has long been the gold standard in LT (132), machine perfusion (MP) preservation has been actively studied and increasingly implemented in clinical practice as a means to overcome hepatic ischemia-reperfusion injury (133). Advances in organ preservation techniques, such as normothermic machine perfusion (NMP), allow for better maintenance of liver grafts by providing oxygen and nutrients during cold storage. NMP has been shown to reduce the extent of cold ischemia-induced damage to LSECs and decrease susceptibility to IRI (134). Optimizing preservation techniques based on the type of ischemia and cell-specific vulnerabilities could further enhance graft quality. The clinical application of MP in liver transplantation has expanded notably in recent years (135). NMP and hypothermic oxygenated perfusion (HOPE) are increasingly implemented, particularly for extended criteria donors (ECD) and donation after circulatory death (DCD) grafts. However, biomarkers for viability assessment (transplant suitability evaluation) after machine perfusion have not yet been established, and no consensus exists on this matter. In addition, most randomized controlled trials and prospective studies have focused on short-term outcomes within 90 days to one year post-transplant (136–138). Although a recent paper reported the feasibility of HOPE in DBD or DCD grafts in the context of long-term outcomes (139), further accumulation of cases is warranted to secure the favorable long-term impacts, such as 5-year survival, chronic biliary complications, and immunomodulatory effects. Nevertheless, MP will be a promising treatment for mitigating ischemia-reperfusion injury in liver transplantation.

## 6 Conclusion

The success of liver transplantation depends on minimizing cell death and controlling the inflammatory response triggered by ischemia-reperfusion injury. While cell death pathways, including apoptosis, necrosis, necroptosis, pyroptosis, panoptosis, ferroptosis, autophagy, and netosis contribute to liver damage, a comprehensive understanding of their specific roles remains critical. By elucidating the interactions between different forms of cell death and the immune response, researchers can develop targeted therapies to protect liver grafts and improve transplantation outcomes. Innovations in therapeutic strategies, combined with advancements in organ preservation, hold the promise of enhancing graft survival and reducing the burden of liver transplantation on patients and healthcare systems.
